# Chick chorioallantoic membrane model to investigate role of migrasome in angiogenensis

**DOI:** 10.52601/bpr.2023.230021

**Published:** 2023-10-31

**Authors:** Cuifang Zhang, Helen He, Shuyao Yin, Mingyi Gao, Li Yu

**Affiliations:** 1 The State Key Laboratory of Membrane Biology, Tsinghua University-Peking University Joint Centre for Life Sciences, Beijing Frontier Research Center for Biological Structure, Beijing 100084, China; 2 Xiamen Cardiovascular Hospital, Xiamen University, Xiamen 361005, Fujian, China

**Keywords:** Angiogenesis, Chorioallantoic membrane (CAM), Chick CAM model

## Abstract

The development of the vascular system is essential for embryonic development, including processes such as angiogenesis. Angiogenesis plays a critical role in many normal physiological and pathological processes. It is driven by a set of angiogenic proteins, including angiogenic growth factors, chemokines, and extracellular matrix proteins. Among various animal model systems, the chorioallantoic membrane (CAM), a specialized and highly vascularized tissue of the avian embryo, has proven to be a valuable tool for analyzing the angiogenic potential of candidate cells or factors. In this protocol, we provide detailed procedures for establishing the CAM model to evaluate the function and mechanism of migrasomes in embryonic angiogenesis. This includes the CAM nylon mesh assay and CAM *ex vivo* sprouting assay to assess CAM angiogenesis, as well as the observation, purification, and delivery of migrasomes. Additionally, we describe the generation of T4-KO-mCherry-KI embryos using the CRISPR system within the CAM tissue to investigate the role of migrasomes in angiogenesis.

## INTRODUCTION

Angiogenesis is a progressive process in which new blood vessels are generated from pre-existing vasculature. To analyze the mechanisms underlying physiological and pathological angiogenesis, numerous *in vivo* angiogenic assays have been established using different species of laboratory animals, including mammals, birds, and zebrafish. In this manuscript, we will focus on major models of angiogenesis in the chick embryo. The use of chick embryo models for angiogenic studies is facilitated by a specialized respiratory tissue known as the chorioallantoic membrane (CAM). This structure rapidly expands, generating a rich vascular network that provides an interface for gas and waste exchange (Deryugina and Quigley 2008). The CAM is a relatively simple, quick, and low-cost model for studying tissue grafts, tumor growth and metastasis, drug delivery, toxicological analysis, and angiogenic and anti-angiogenic molecules. Moreover, because the chick embryo is naturally immunodeficient, it can receive transplantations from different tissues and species without eliciting immune responses (Ribatti 2017).

During chick embryo development, the allantois appears at approximately embryonic day 3.5 (E3.5d). The allantoic vesicle develops rapidly starting from embryonic day 4 (E4d) and reaches its peak at embryonic day 10 (E10d). This allantois fuses with the adjacent mesodermal layer of the chorion to form the CAM. The CAM vascularization undergoes three phases of development: the first phase involves sprouting and fusion to form the primary capillary plexus; the second phase, instead of sprouting, is characterized by predominantly intussusceptive angiogenesis, promoting capillary growth; and in the last phase, the growing pillars increase to form capillary meshes. Because immature blood vessels scattered in the mesoderm grow very rapidly until day 8–10 and give rise to a capillary plexus, we selected CAM samples at embryonic day 8–10 (Chen *et al.* 2021; Deryugina and Quigley 2008; Ribatti 2017).

Migrasomes are newly discovered organelles that form at the tips or intersections of retraction fibers (RFs). When a cell migrates, numerous retraction fibers are pulled out from the trailing edge of the cell, and large vesicles named migrasomes grow on these retraction fibers. These structures are released into the extracellular environment or taken up by surrounding cells, mediating the release of cytoplasmic contents and facilitating intercellular communication (Ma *et al.* 2015).

During zebrafish embryonic development, migrasomes are enriched with the chemokine CXCL12, which acts as a chemoattractant to guide the migration of dorsal forerunner cells (Jiang *et al.* 2019). Furthermore, migrasomes have been shown to mediate the lateral transfer of mRNA among cells (Zhu *et al.* 2021). Therefore, migrasomes have been proposed as organelles for cell–cell communication. However, research on the physiological functions of migrasomes is still in its early stages.

Based on recent advances in migrasome research and animal model studies of angiogenesis, we describe a series of assays using the CAM model to investigate the physiological functions of migrasomes in embryo development (Zhang *et al.* 2022). This may help expand the application of the chick CAM model in the study of angiogenesis.

### Overview of the protocol

Given the nascent stage of exploration into the physiological functions of migrasomes, it remains uncertain whether migrasomes play a role in angiogenesis during embryonic development (Tan *et al.* 2023; Zhang *et al.* 2022). The CAM, serving as an animal model for the study of blood vessel formation, offers distinct advantages which we describe below. However, it is noteworthy that chicken embryos, belonging to the avian species and evolutionarily distant from mammals, render the chicken embryo animal model relatively specialized and niche in nature (Fishman *et al.* 2019). Consequently, the repertoire of established experimental systems related to chicken embryos remains rather limited. Therefore, it is imperative to establish and optimize numerous new methodologies utilizing the CAM model. In this paper, we present protocols primarily tailored to the research requirements of migrasomes, comprising five integral components: gene editing in chick embryos, observational systems in the CAM, migrasome purification and delivery in the CAM, and a CAM angiogenesis assessment system.

### Advantages of the CAM model

The CAM model is a widely used experimental platform in diverse biomedical research disciplines, offering numerous advantages (Nowak-Sliwinska *et al.* 2014). It provides easy access to the availability of chick embryos and the straightforward accessibility of the CAM for manipulation and observation. Its high vascularity ensures a robust blood supply, making it ideal for studying angiogenesis, tumor growth, and drug delivery. Additionally, its transparency allows direct real-time monitoring, enabling observations of processes such as blood vessel formation, graft integration, and drug diffusion.

Furthermore, the CAM model offers an environment that closely resembles either *in vivo* or *ex vivo* conditions, facilitating the investigation of physiological processes and interactions with various substances or materials. Its cost-effectiveness makes it a financially sound choice for large-scale research projects compared to alternative animal models. Lastly, the ethical consideration of using chick embryos aligns with regulations on animal welfare, reducing the necessity for other vertebrate animals in experiments

### Limitations of the CAM model

The CAM model is a widely used experimental model in angiogenesis research due to its accessibility, low cost, and ease of use. However, it does have some disadvantages that should be considered. First, species specificity: the CAM model is specific to avian species. This restricts its application when studying mammal-specific angiogenesis mechanisms. Second, limited availability of reagents, such as antibodies. Third, it is relatively difficult to genetically modify the CAM. Researchers should carefully weigh its advantages and disadvantages and complement their findings with other models.

## SUMMARIZED PROCEDURE

All institutional and national guidelines for the care and use of laboratory animals were followed. All animal studies were conducted according to the guidelines from the Animal Care and Use Committee of Tsinghua University and Xiamen University.

### Observation of migrasome formation in CAM *in vivo*

1 A square hole was cut in the eggshell of embryonic day 9 (CAM9d).

2 CAM was stained by wheat germ agglutinin (WGA).

3 Visualize CAM in a glass holder under a confocal microscope.

### Migrasome purification in CAM9d

1 Migrasome purification was performed by iodixanol sucrose density-gradient centrifugation using an Optiprep kit (LYSISO1, Sigma-Aldrich). CAMs were isolated from eight embryonic 9-day old chicken embryos (CAM9d), and then subjected to mechanical mincing.

2 The chopped-up CAMs were shakingly digested with collagenase II and trypsin.

3 The samples were centrifuged to remove the cell bodies and cell fragments, and finally centrifuged to collect crude migrasome sediment.

4 The crude migrasome sediment was fractionated at 150,000 *g* in a multistep Optiprep dilution gradient to get the purified migrasome fraction.

### Migrasome delivery and CAM angiogenesis assay

1 Open the window of the eggshell of embryonic day 9 (CAM9d) so that the CAM can be observed.

2 Preparation of Matrigel- or agarose-embedded migrasomes.

3 The congealed mixture embedded in migrasomes was placed on the designated side of the CAM *in vivo*.

4 Examine and visualize the newly formed vessels at the position of migrasome delivery.

### The CAM nylon mesh assay

1 Prepare the nylon meshes (cut into 4.41 mm^2^ per piece).

2 Migrasome embedded by a Matrigel-collagen I mixture was placed onto the nylon mesh for polymerization.

3 Polymerized meshes were placed onto the outer region of CAM8d and incubated.

4 The newly formed vessels were examined and visualized with a photo microscope.

### The CAM *ex vivo* sprouting assay

1 Preparations of Matrigel-Collagen I matrix mixture on 35 mm confocal dishes.

2 Dissected CAMs (about 1 mm^2^) from E8d were harvested and embedded in the matrix.

3 After 24 h, drops of soft agarose with or without migrasomes were added on opposite sides of the CAM.

4 Visualize and analyze ECs sprouting from CAMs.

### Generation of T4-KO-mCherry-KI embryos by the CRISPR system

1 TSPAN4-Gallus sgRNA was designed and inserted into the sgRNA expression cassettes of the pUC57 vector.

2 Evaluation of the TSPAN4 knockout efficiency by mCherry knock-in system.

3 Generation of the TSPAN4-KO chick embryos by microinjection of CRISPR system mRNA mixture (TSPAN4-sgRNAs, Cas9 and mCherry-KI) into chick embryos and then electroporation.

4 Detection of TSPAN4 Knockout efficiency by FACS and IF.

## PROCEDURE

### Preparations of fertilized chick eggs [TIMING 18 h–15 d]

1 Fertilized SPF eggs (Variety: White Leghorn; Cleanliness: SPF, cleaned and disinfected with ultraviolet light; Storage: stored in a constant temperature chamber at 14 ± 2 °C, no more than ten days) were bought from Beijing Boehringer Ingelheim Vital Biotechnology Co., Ltd.

2 The eggs were incubated in a hatching incubator at 37.5 °C with 60%–70% humidity. Eggs were turned every five minutes.

3 All SPF chick embryos used in the study were incubated from 18 hours to 15 days (E15d or CAM15d).

### Observation of migrasome formation in CAM *in vivo* [TIMING 5 h]

Because opening the window will disturb the temperature, humidity, and atmospheric pressure of the chicken embryo in the egg, it should be ensured that the chicken embryo can still survive when we observe the CAM. After numerous assay optimizations, we have successfully resolved the issue and established the assay as follows.

1 A square hole (about 1 × 1 cm^2^) was cut in the eggshell of embryonic day 9 (CAM9d).

2 WGA was diluted in 1× PBS (1:500, 200 µL 1× PBS) and then this mixture was added to the top of the CAM.

3 Staining for 20 min at 37.5 °C in a humidified incubator with 70% humidity.

4 The egg was placed on a holder with the hole in direct contact with a cover glass, so that the weight of the egg held the CAM in tight contact with the cover glass.

5 Last, the CAM was visualized under a confocal microscope for about 4 h.

### Migrasome purification [TIMING 6 h]

Migrasome purification was performed by iodixanol sucrose density-gradient centrifugation using an Optiprep kit (LYSISO1, Sigma-Aldrich), the detailed procedure is shown below.

1 CAMs were isolated from eight E9d chicken embryos, and then subjected to mechanical mincing.

2 The chopped-up CAMs were shakingly digested with collagenase II for 30 min at 37 °C.

3 Centrifugation, 1000 *g*, 5 min, got the sediment.

4 The above sediment was treated with trypsin for another 20 min at 37 °C.

5 The samples were centrifuged at 1,000 *g* for 5 min at 4 °C to remove the cell bodies and get supernatant.

6 The above supernatant was followed by 4,000 *g* for 20 min at 4 °C to remove the cell fragments and get the supernatant.

7 The above supernatant was finally centrifuged at 20,000 *g* for 20 min at 4 °C to get the sediment.

8 The pellet containing the crude migrasome fraction was resuspended and lysed in extraction buffer (Sigma-Aldrich) and then fractionated at 150,000 *g* for 4 h at 4 °C in a multistep Optiprep dilution gradient. The detail gradient was: 3%, 5%, 8%, 12%, 16%, 19% (sample), 22.5% and 27%.

9 After centrifugation, the fraction formed, and the migrasome was enriched at fractions 3–5.

10 Fractions were collected and added to 500 μL PBS. Centrifugation was then performed at 20,000 *g* for 30 min at 4 °C to collect migrasome.

11 The pellet was washed once with PBS and centrifuged at 4 °C, 2,000 *g* for 10 min.

12 The supernatant was collected and centrifuged at 4 °C, 20,000 *g* for 30 min to obtain migrasomes for microscope observation or delivery.

### Migrasome delivery onto CAM [TIMING 3 d]

1 Fertilized chicken eggs were incubated at 37.5 °C for 9 days and then opened so that the CAM could be observed.

2 Preparation of migrasome embedded matrix. Firstly, 30 µg of purified migrasomes (from CAM of chicken embryos at E9d) were resuspended in 3 μL 1× PBS.

3 Resuspended migrasomes were mixed with5 µL 2% low-melting-point agarose. As the control, 30 µg cell body isolated from CAM was mixed with 5 µL 2% low-melting-point agarose.

4 The congealed mixture was placed on the designated side of the CAM *in vivo* for about three days.

5 Three days later, numerous allantoic vessels developed in a "spoked-wheel" pattern. Then images were immediately captured by a stereoscope or confocal microscope. The density or number of newly formed capillaries in the CAM was quantified with Image J v1.8.0.112 or Image-Pro Plus software (Media Cybernetics).

### The CAM nylon mesh assay [TIMING 5 d]

Since directly observing capillary growth towards migrasomes may be prone to artifacts, we carried out the Matrigel invasion assay, in which a nylon mesh coated by a collagen I-Matrigel matrix with or without migrasomes is placed on top of the CAMs, and neovascularization occurs upward and against gravity to invade the matrix. New blood vessels are induced to grow upright into the matrix from the underlying CAM, and therefore can be clearly discriminated from the background vascular network, providing a clear advantage for angiogenesis scoring. This represents a modification of the CAM angiogenesis method to offer a straightforward approach to quantifying angiogenesis.

1 Preparation of nylon meshes. The nylon meshes (TS207-050, SK, pore size 300 µm, China) were cut into 4.41 mm^2^ pieces, then autoclaved for 10 min and vacuum-dried.

2 Open the window of the eggshell on chick embryonic 8d to expose the CAM.

3 Prepare the nylon mesh-matrix-migrasome polymerization mixture. 10 µL Matrigel and 5 µL collagen I mixed either with 5 uL cell body from CAM (as control) or with 5 µL migrasomes (10 µg/µL) were placed onto the nylon mesh and incubated at 37 °C for 30 min and at 4 °C for 1 h for polymerization.

4 The polymerized mixture was placed onto the outer region of CAM8d and incubated for five days.

5 The newly formed vessels were examined and visualized with a photo microscope (Leica EZ4W) or by Nikon Ti2-E.

6 The density or number of newly formed capillaries in the CAM was quantified with Image J v1.8.0.112 or Image-Pro Plus software (Media Cybernetics).

### CAM *ex vivo* sprouting assay [TIMING 3–6 d]

1 Depolymerization of matrigel in 4 °C

2 Matrix containing 150 µL Matrigel and 30 µL collagen I was mixed on ice and then added into 3.5 mm confocal dishes and incubated at 37 °C for 1 h for polymerization.

3 Dissected CAMs (about 1 mm^2^) from CAM9d were harvested and embedded in the matrix for 24 h without any medium in it.

4 24 h later, add about 50 μL complete ECM to keep the humidity of CAM in the dish.

**[CAUTION!]** Matrigel-Collagen I Matrix preparation must be on the ice to keep the matrigel liquid state. Before dissected CAMs embedded in the matrix for 24 h, we should not add any buffer or medium in the culture dish to guarantee CAM adherence to the matrix.

5 Drops of low melt agarose with or without migrasomes were added on opposite sides of the CAM for another 2–5 days.

6 After 2–5 days of culture, ECs sprouting from CAMs were visualized by Nikon Ti2-E and analyzed by Nikon NIS-elements.

7 Post-treated CAMs were stained by ASM1 and Actin, then the samples were visualized by Nikon A1 confocal microscopy to assess the role of migrasomes in chemo-attraction of ECs and tip-cell filopodia formation.

### Generation of T4-KO-mCherry-KI embryos by the CRISPR system [TIMING 8 d]

Cas9 nickase and paired sgRNAs can be used to efficiently modify the genome of embryos with minimal off-target damage. This protocol describes the design and construction of paired sgRNAs plasmids, and the preparation of sgRNAs and Cas9 mRNAs for the injection of gastrulating chick embryos (E18h).

#### Part 1: Construction of pUC57-TSPAN4-sgRNA plasmids by the CRISPR system

Cas9 nickase and paired sgRNAs can be used to efficiently modify the genome of embryos with minimal off-target damage. This protocol describes the design and construction of pUC57- and TSPAN4-sgRNA plasmids.

1 Design of paired sgRNA oligos as manual. Order oligos as below.

**[CAUTION!]** For each sgRNA, the 5’-GGN_(19)_GG motif is preferred, and 5’-GN_(20)_GG or 5’-N_(21)_GG are also satisfactory. BLAT or BLAST, the sgRNA target sites in UCSC or ENSEMBL genome browsers to find those with few or no highly related sites in the genome.

For 5’-GGN_(19)_GG motif:


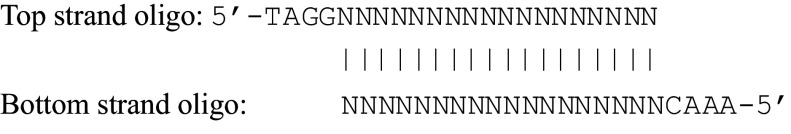


For 5’-GN_(20)_GG motif:


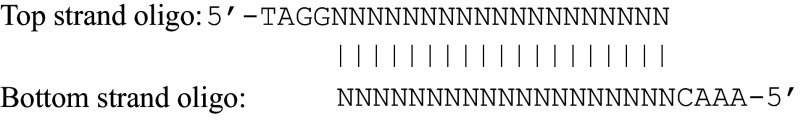


For 5’-N_(21)_GG motif:


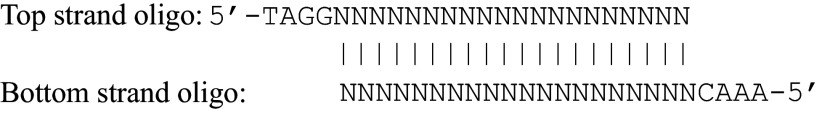


2 Annealing oligos prior to cloning.

4.5 µL top oligo (100 µmol/L),

4.5 µL bottom oligo (100 µmol/L),

1 µL NEB Buffer 2.

Annealing the oligos using a thermocycler with the following program:

95 °C, 5 min; 95 to 85 °C at −2 °C/s; 85 to 25 °C at −0.1 °C/s; hold at 4 °C.

3 Preparation of pUC57-sgRNA plasmid (TSPAN4-KO guide RNA coding sequence was cloned into pUC57-sgRNA vector).

2 µg pUC57-sgRNA,

1 µL CutSmart Buffer,

1 µL BsaI.

Add H_2_O up to 50 µL and incubate at 37 °C for 2 h with occasional shake.

Purify the digestion product using a MinElute PCR Purification Kit.

4 Ligation of annealed oligos with BsaI-digested pUC57-sgRNA.

2 µL annealed oligos prepared from above Step 2,

1 µL (25 ng/µL) digested pUC57-sgRNA,

3 µL 2× Solution I.

Incubate them at 16 °C for 30 min.

5 Transformation and plate on Kan^+ ^ plate (50 μg/mL).

6 Confirm correct insertion of TSPAN4-sgRNA oligos by sequencing using M13-47 primer.

7 Mini-prep pUC57-TSPAN4-sgRNA plasmids using QIAprep Spin Miniprep Kit.

#### Part 2: Transcription of pUC57-TSPAN4-sgRNAs in vitro

1 Ensure that reagents, tubes and tips are RNase-free and that the work is done in a ribonuclease-free environment.

2 Digest paired pUC57-TSPAN4-sgRNA plasmids with Dra I and purified the digestion product.

10 µg paired pUC57-TSPAN4-sgRNA plasmids (5 µg each),

10 µL 10× M buffer,

5 µL Dra I (15 U/µL).

Add H_2_O up to 100 µL and incubate at 37 °C for 3 h with occasional shake.

**[Tip]** Check plasmids were digested completely by gel electrophoresis.

3 *In vitro* transcription of sgRNAs using MEGAshortscript™ Kit.

1 μL T7 10X reaction buffer,

1 μL T7 ATP solution (75 mmol/L),

1 μL T7 CTP solution (75 mmol/L),

1 μL T7 GTP solution (75 mmol/L),

1 μL T7 UTP solution (75 mmol/L),

4 µL purified template (more than 2 µg for plasmids, 700–1000 ng for PCR products),

1 μL T7 enzyme mix,

10 µL of transcription volume is OK.

Incubate the reaction at 37 °C for 4–6 h in a water bath or Thermocycler (Set the hot lid to 50 °C).

Add 1 μL TURBO DNase and incubate at 37 °C for 15 min to remove the DNA template.

4 Purify the TSPAN4-sgRNAs by MEGAclear™ Kit according to the manufacturer’s instructions.

5 Aliquot and store at −80°C. The TSPAN4-sgRNAs are stable for one year without freeze-thaw cycles.

#### Part 3: Transcription of Cas9 in vitro

1 Ensure that reagents, tubes and tips are RNase-free and that the work is done in a ribonuclease-free environment.

2 Digest Cas9 plasmid with Age I and purify the digestion product.

10 µg Cas9,

10 µL NEB Buffer 1,

4 µL Age I.

Add H_2_O up to 100 µL and incubate at 37 °C for 3 h with occasional shake.

3 Add 4 µL RNAsecure and incubate at 60 °C for 10 min in a thermomixer.

4 Digest Cas9 plasmid with Age I and purify the digestion product.

5 Check for complete digestion of the plasmid by electrophoresis, loading 2 µL in 1% agarose gel.

6 Purify and elute the digestion product using the MinElute PCR Purification Kit.

7 *In vitro* transcribe Cas9 using mMESSAGE mMACHINE^®^ T7 Ultra Kit according to the manufacturer’s instructions.

8 Purify the Cas9 mRNA by RNeasy Mini Kit according to the manufacturer’s instructions.

9 Assess sgRNA yield using the One Drop OD-1000+ Spectrophotometer (or equivalent) and sgRNA quality by gel electrophoresis.

10 Aliquot and store at −80°C. Cas9 mRNA is stable for one year without freeze-thaw cycles.

#### Part 4: Generation of T4-KO-mCherry-KI plasmid and mRNA

Since there are no commercially available antibodies to detect TSPAN4, to evaluate the TSPAN4 knockout efficiency, mCherry was inserted into TSPAN4 at the position targeted by the sgRNA by mCherry-KI plasmid: T4-KO-mcherry-KI plasmid (Zhang *et al.* 2022).

1 Plasmid extraction and purification.

2 Got T4-Gallus-KO-mcherry-KI mRNA as above mentioned.

#### Part 5: Microinjection of the mRNA mixture into chick embryonic 18 h

1 Cut a square hole (0.6 × 0.6 cm^2^) in the eggshell of gastrulating chick embryos (E18h).

2 Thaw aliquots of the Cas9 mRNA, TSPAN4-KO-sgRNAs, and T4-KO-mcherry-KI mRNA on ice. Dilute the above mRNA mixture with EmbryoMax® Injection Buffer to a concentration of 30 ng/μL Cas9 mRNA, 10 ng/μL TSPAN4-KO-sgRNAs mRNA, 10 ng/μL T4-KO-mcherry-KI mRNA, the total volume is 10 μL. Pipette the mixture up and down several times.

3 Centrifuge at 4 °C for 1 min at top speed, and carefully transfer the above 10 μL supernatant into chick embryos at Hamburger Hamilton stage 4 (HH4, embryonic 18 h) using a glass capillary with a tip diameter of 0.1 mm.

4 Chick embryos were then electroporated using previously described techniques (Sauka-Spengler and Barembaum 2008).

5 The hole was covered by Parafilm and the chick embryos were incubated at 37.5 °C in the air under 70% humidity.

6 Knockout effciency were confirmed by direct FACS for mCherry-positive signal sorting and by *in vivo* imaging for mCherry-positive signal detection.

## ANTICIPATED RESULTS

**Figure 1:**
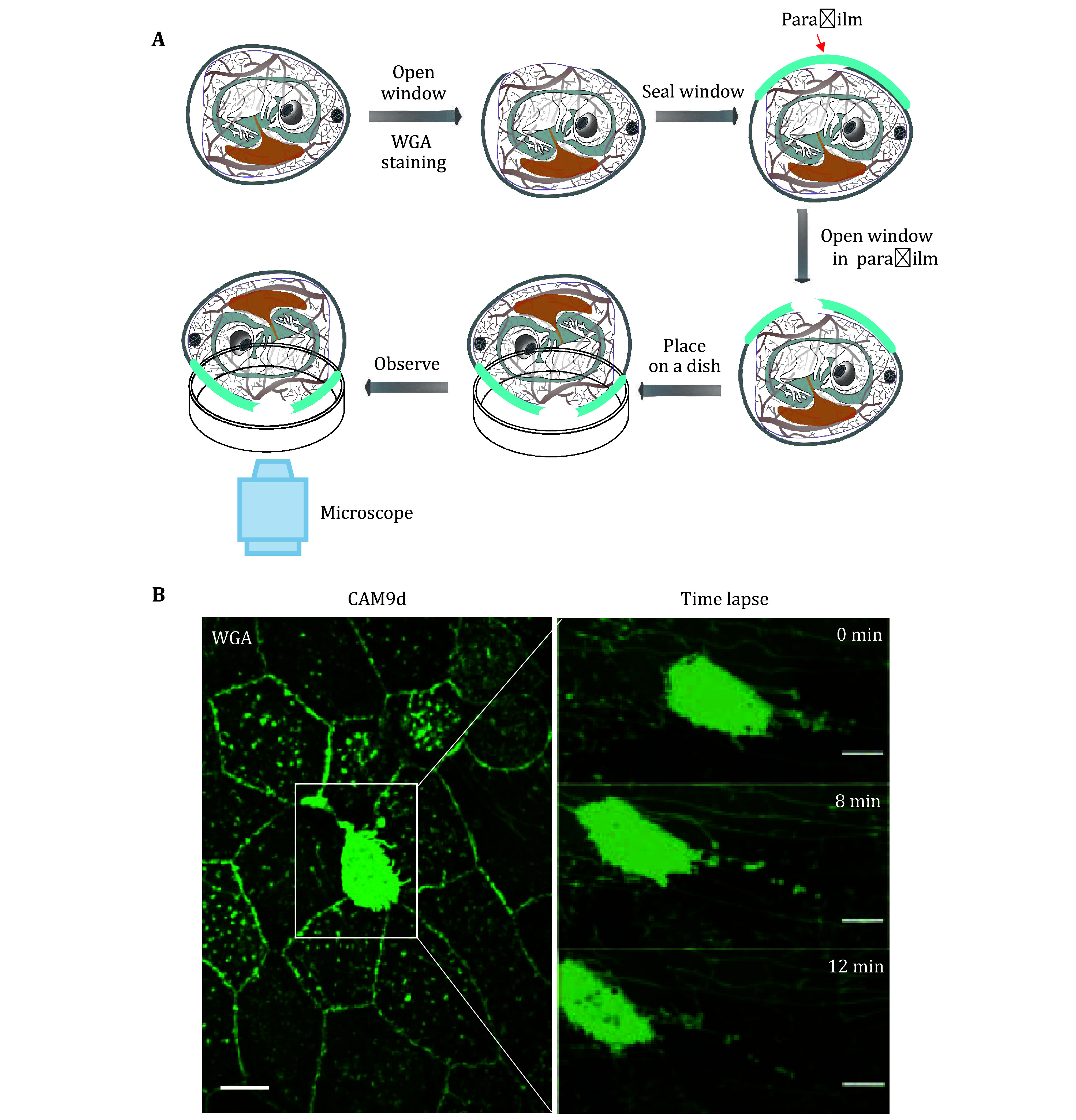
Observation of migrasome formation in the CAM *in vivo*. **A** Diagram of migrasome observation procedure in CAM. **B** Images of migrasomes formation of WGA_high_ cell in CAM9d *in vivo* level. Left: confocal image of CAM stained by WGA *in vivo* level; scale bar, 8 µm. Right: time-lapse of migrasomes formation in CAM9d *in vivo* level; scale bar, 5 µm

1 Following the protocol, we first designed an imaging protocol. Figure 1A shows the detailed operating process. The CAM stained by WGA was imaged and the migrasome formation by WGA_high_ has been shown in Fig. 1B.

**Figure 2:**
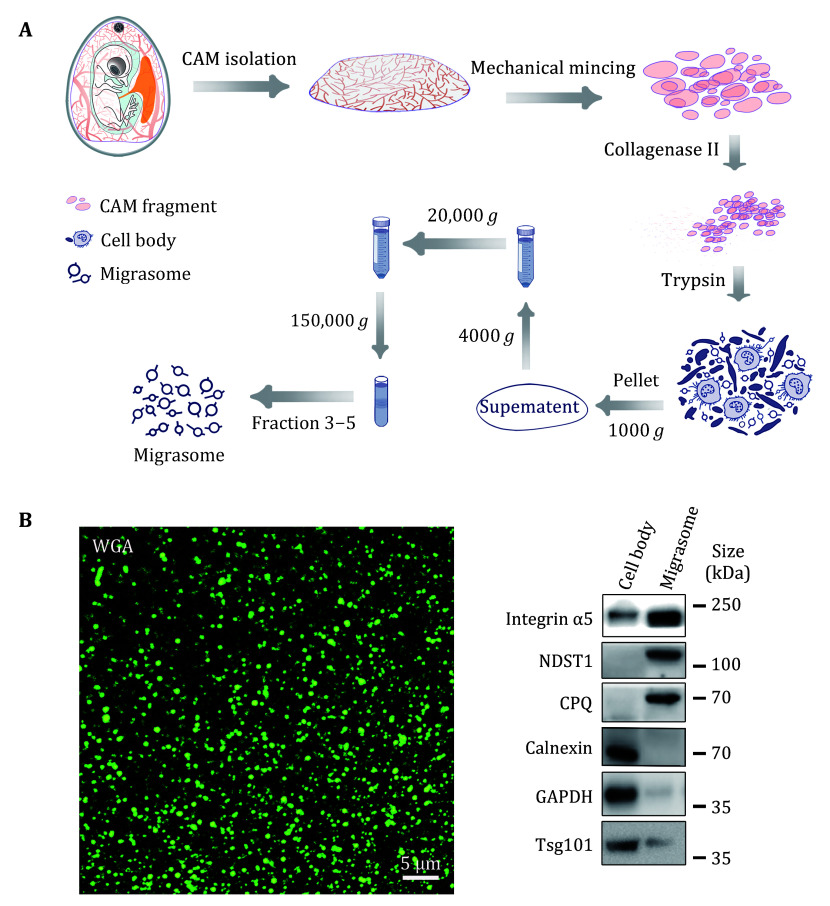
Migrasome purification from CAM9d tissue. **A** Diagram of migrasome from the procedure from CAM9d tissue. **B** Migration purity identification. Left panel: images of migrasomes stained by WGA purified from CAM9d; scale bar, 5 µm. Right panel: Western blot analysis of isolated CAM9d migrasomes with the indicated antibodies

2 Since we have found the migrasome in CAM tissue, to detect the function of migrasome, we established the protocol for migrasome isolation and purification from CAM tissue. Figure 2A shows the detailed operating process. The purity of the isolated migrasomes in Fig. 2B was analyzed by IF and by western blot for various migrasome markers we reported previously (Zhang *et al.* 2022). We found that the isolated migrasomes have the characteristic morphological features of migrasomes, and migrasome markers are highly enriched in the preparation.

**Figure 3:**
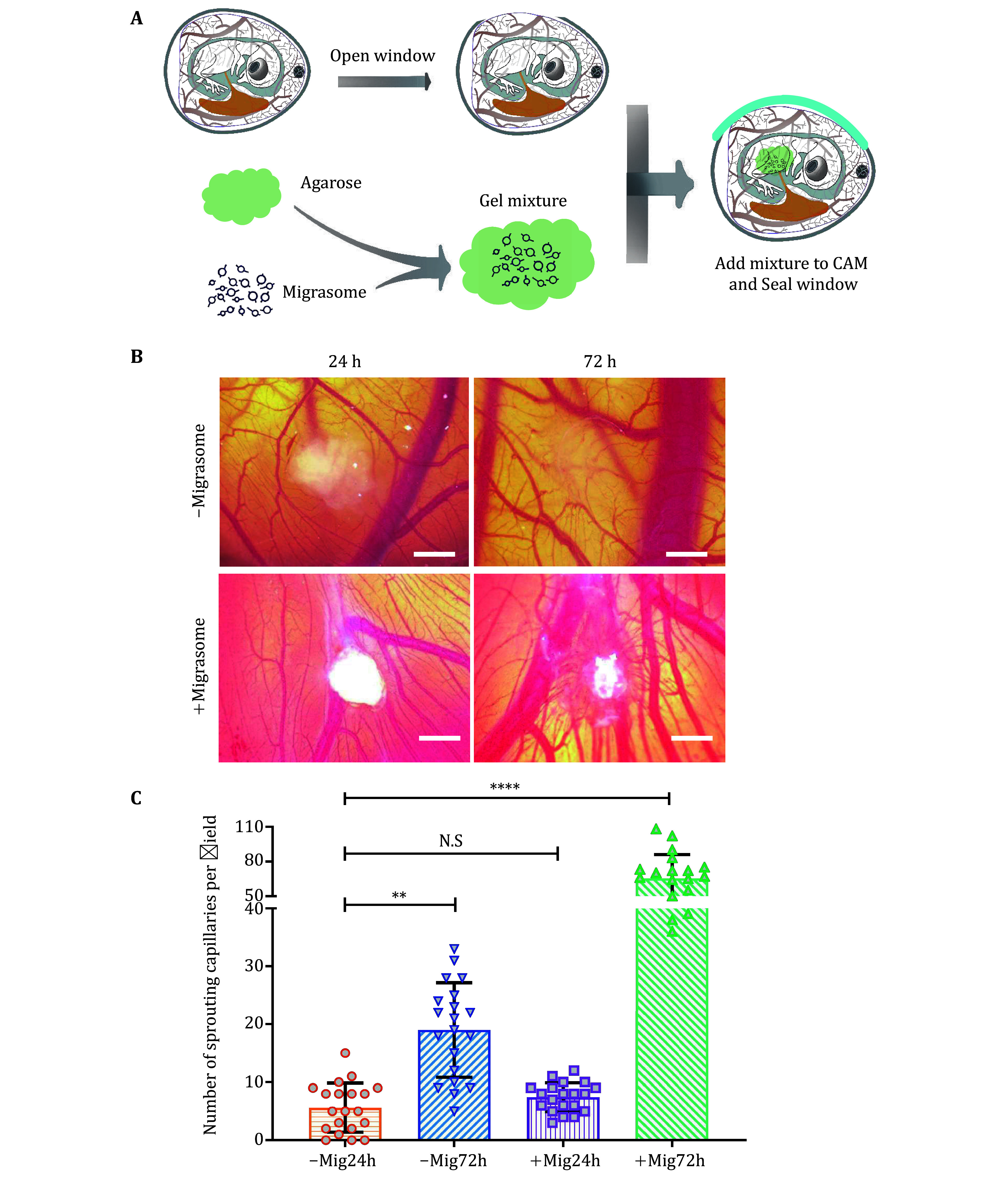
Migrasome delivery onto CAM9d. **A** Diagram of migrasome delivery onto CAM9d. **B** Migrasomes were delivered to CAM9d by mixing them with low-melting-point agarose. After 24 h and 72 h, CAMs were visualized by stereomicroscopy. Scale bar, 500 µm. **C** Quantification of sprouting capillaries number per field. Data are presented as mean ± SEM, *n* = 20 fields from three independent experiments. *P* values, two-tailed, unpaired *t*-test. ^**^P < 0.01; N.S, no significance; ^****^P < 0.0001

3 After we got the high-purity migrasome from CAM tissue, we delivered the migrasome to CAM to detect its function in angiogenesis. The detailed operating process is shown in Fig. 3A. The migrasome embedded into low melting agarose was added into CAM9d，three days later, We found that migrasomes significantly promoted capillary formation in this assay in contrast to the control (Figs. 3B and 3C).

**Figure 4:**
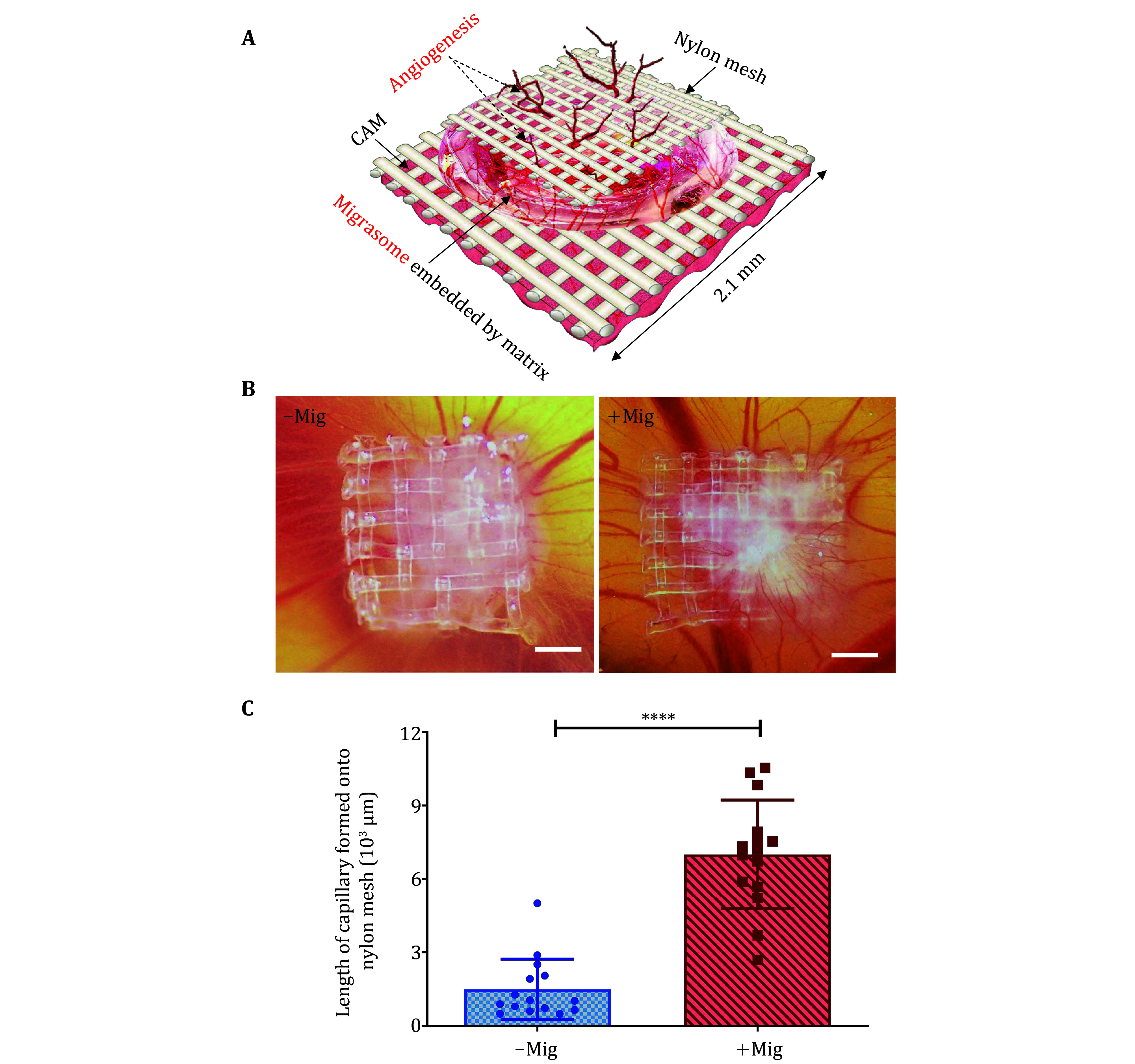
The CAM nylon mesh assay. **A** Diagram of migrasome CAM nylon mesh assay. **B** Migrasomes were delivered to CAM9d by nylon mesh, and newly formed capillaries on nylon mesh were visualized when the migrasome was added. Scale bar, 500 µm. **C** Newly formed capillaries onto nylon mesh were quantified. Error bar, ±SEM; *n* = 15 fields from three independent experiments; *P* values, two-tailed, unpaired *t*-test, *P* < 0.0001

4 Since the migrasome embedded by agarose brings more or less nearby vessels hypoxia and gives the impression of a higher capillary density. We established an invasion assay named a nylon mesh assay where the growth of new vessels occurs upward and against gravity by invading a new matrix and independent of the plane of the CAM, this protocol may be far more desirable and less prone to artifacts. Figure 4A shows the diagram of how to operate the process. Figures 4B and 4C show the results when nylon mesh coated by a collagen I–Matrigel matrix with migrasomes was placed on top of the CAMs, the neovascularization upward and against gravity significantly promoted capillary formation contrast to the matrix without the migrasome.

**Figure 5:**
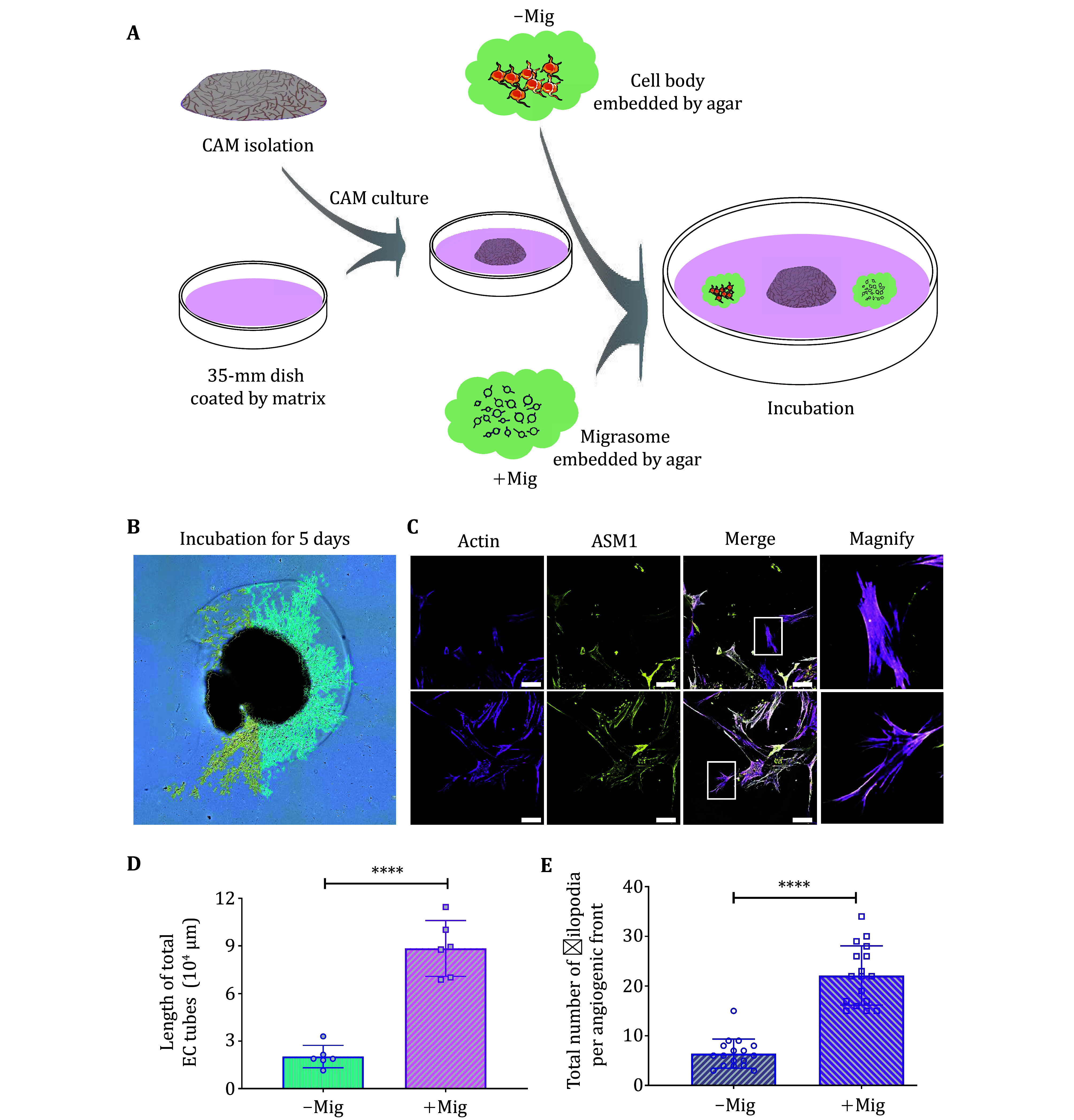
The CAM *ex vivo* sprouting assay. **A** Diagram of CAM *ex vivo* sprouting assay. **B** CAM9d was incubated onto matrige-collagen for five days, then was visualized by stereomicroscopy and analyzed by Nikon NLS element to asses EC sprouting and tube formation. Scale bar, 500 µm. –Mig: only cell body embedded by agar, without migrasome embedded. +Mig: within migrasome embedded. **C** Sample from Panel B was stained by filopodia marker Actin and ASM1, then imaged by confocal microscope. Scale bar, 20 µm. The boxed area is magnified on the right. **D** Quantification of Panel B in terms of EC tube length. Error bar, ±SEM; *n* = 6 CAM leaflets from six independent experiments; *P* values, two-tailed, unpaired *t*-test, *P* < 0.0001. **E** Quantification of the numbers of filopodia from Panel C. Error bar, ±SEM; *n* = 17 fields from six independent experiments; *P* < 0.0001

5 Another assay to estimate the pro- or anti-angiogenic ability of the migrasome is the CAM *ex vivo* sprouting assay. Figure 5A shows the detailed operating process. We found that five days after adding the soft agarose, there were many cells on the side of the CAM close to the migrasome-containing agarose, but not on the side close to the control agarose. Cells on the migrasome side migrated much further from the edge of the CAM compared with cells on the control side. Moreover, there were significantly more EC sprouting events and elongated filopodia on the tip cell marked by ASM1 and actin on the migrasome side than on the control side (Figs. 5B–5E).

**Figure 6:**
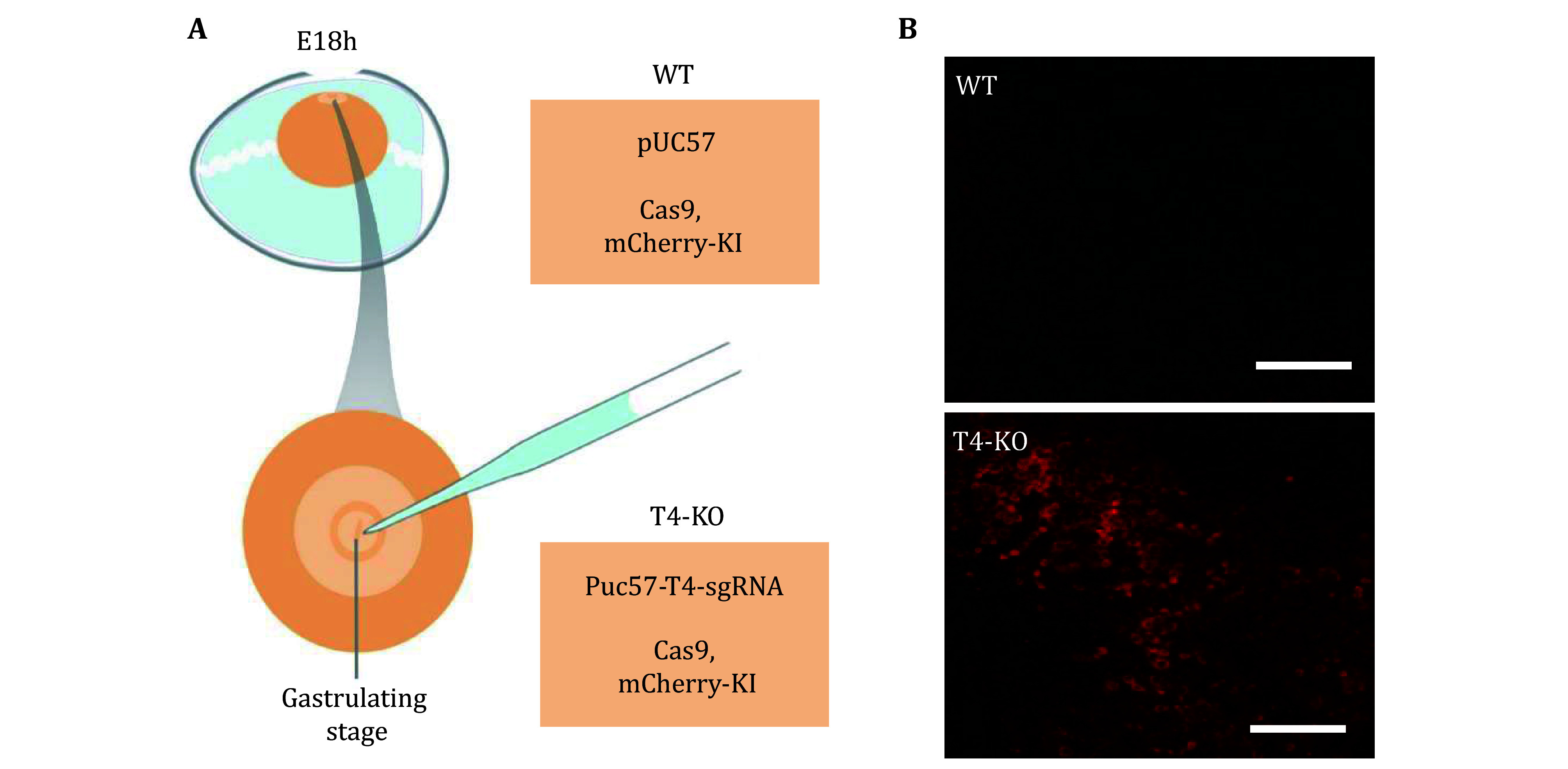
Generation of T4-KO-mCherry-KI embryos by the CRISPR system. **A** Diagram of strategy for generating mosaic knockout chicken embryos. **B** TSPAN4-KO efficiency monitored by expression of mCherry. Scale bar, 20 µm

6 TSPAN4 is essential for the migrasome formation. To detect whether the migrasome elimination decreases angiogenesis in CAM, we established the assay shown in Fig. 6A about knocking out TSPAN4 in the early stage of chicken embryonic development. Knockout of TSPAN4 was achieved by using CRISPR/Cas9. The knockout efficiency is indicated by mCherry expression, which occurs only in cells where TSPAN4 has been successfully knocked out. Figure 6B shows that TSPAN4 knockout efficiency is more than 70%.

## MATERIAL

### Reagents

#### Fertilized SPF eggs and CAM observation

• Fertilized SPF eggs (variety: White Leghorn; cleanliness: SPF) were bought from Beijing Boehringer Ingelheim Vital Biotechnology Co., Ltd

**[CAUTION!]** All experiments using animals must be carried out according to relevant governmental and institutional regulatory guidelines.

• WGA (wheat-germ agglutinin) was from Life Technologies (W11261, Carlsbad, USA)

#### Migrasome purification from CAM

• Phosphate buffered saline was from Cytiva HyClone (SH30256.01, Marlborough, USA)

• Collagenase Type II powder was from Gibco (17101-015, Carlsbad, USA)

• 0.25% Trypsin + 0.02% EDTA solution was from Cienry (CR-25200, Hangzhou, China)

• The Lysosome isolation kit was from Sigma-Aldrich (LYSISO1-1KT, Shanghai, China)

• The anti-Integrinα5 antibody was from Cell Signaling Technology (4705S, Massachusetts, USA). The antibody against GAPDH was from Proteintech (60060004-1-IG, Rosemont, USA). The antibody against NDST was from Santa Cruz Biotechnology (sc-374529, Dallas, USA). The antibody against CPQ was generated by Sigma (HPA023235, Shanghai, China). The antibody against TSG101 was from Abcam (ab125011, Cambridge, USA). The antibody against Calnexin was from Abcam (ab22595, Cambridge, USA). Goat-Anti-Mouse Ig Human ads-HRP (Southern Biotech), cat. no. 1010-05, RRID: AB_2728714, 1:5000 dilution. Goat-Anti-Rabbit Ig Human ads-HRP (Southern Biotech), cat. no. 4010-05, RRID: AB_2632593 1:5000 dilution

#### Migrasome delivery onto CAM9d

• Low melting agarose II was from AMRESCO (0815-25G, USA)

• Matrigel Basement Membrane Matrix was from Corning (356234, New York, USA)

• Collagen I coating solution was from Sigma-Aldrich (122-20, USA)

• The nylon mesh was from SK (TS207-050, SK, pore size 300 µm, China)

#### CAM ex vivo sprouting assay

• Penicillin&Streptomycin solution was from GENOM (GNM15140, Hangzhou, China)

• GlutaMAX™ I (100×) was from Gibco (35050-061, Carlsbad, USA)

• 4% Paraformaldehyde was from Dingguo Changsheng Biotechnology (ar-0211, Beijing, China)

• GlutaMAX™ I (100×) was from Gibco (35050-061, Carlsbad, USA)

• The anti-Actin antibody was from Thermo Fish (MA5-11869, USA)

• The ASM1 (Alpha-Smooth Muscle Actin) antibody was from Thermo Fish (MA1-06110, USA)

#### Generation of T4-KO-mCherry-KI embryos by the CRISPR system

• pUC57-sgRNA (Addgene 51132, promoter: T7)

• mMESSAGE mMACHINE® T7 Ultra Kit (Ambion, AM1345)

• MEGAshortscript™ Kit (Ambion, AM1354)

• RNeasy Mini Kit (QIAGEN, 74104)

• MEGAclear™ Kit (Ambion. AM1908)

• RNA*secure*™ Reagent (Ambion. AM7005)

• QIAprep Spin Miniprep Kit (QIAGEN, 27104)

• MinElute PCR Purification Kit (QIAGEN, 28004)

• Bsa I (NEB, R0535S)

• Dra I (TaKaRa, D1037A)

• Solution I (TaKaRa, 6022Q)

• PMSG (Sansheng, China. 50 IU/mL in normal saline. Aliquot and store at –80 °C)

• EmbryoMax^®^ Injection Buffer (Millipore, MR-095-10F)

• Phenol (Tris-saturated), Chloroform and alcohol

• T-Vector pMD™19 (Simple) kit (TaKaRa, 3271)

• 2xRealStar green power mixture was from Gibco (A311-01, Carlsbad, USA)

• TaKaRa MiniBEST Universal RNA Extraction kit was from TaKaRa (9767, Kusatsu, Japan)

• Endofree plasmid Midi kit was from CWBIO (CW2105S, Taizhou, China)

• TIANgel Midi Purification kit was from Tiangen (DP209-02, Beijing, China)

## EQUIPMENT

• The 35-mm cover glasses confocal dish (Biosharp, BS-20-GJM, USA)

• Centrifuge (RT and 4 °C)

• Vortex

• One Drop OD-1000+ Spectrophotometer

• Thermocycler

• Thermomixer

• Water bath

• Parafilm (Bemis, PM-996, USA)

• 0.22-μm centrifugal filter (Millipore, SLGP033NS)

• 1.5-mL microtube (Axygen, cat. no. MCT-150-C)

• 15-mL Centrifuge tube (YUEYI, cat. no. YB0019-15)

• 50-mL Centrifuge tube (YUEYI, cat. no. YB-50D)

• Pipettes: 25, 10, 5 and 1 mL

• Images are acquired by FV31S-SW V2.3.1 (Olympus), Velocity 6.3.1 (PerkinElmer), Andor spinning disk confocal dragonfly200 (Andor technology Ltd, Belfast, UK), Nikon A1 confocal (HD25RSi LSCM), Leica EZ4W stereomicroscope

• NIS-Element AR analysis was used for cell tracking. Image J v1.8.0.112 was used for the analysis of the number of sprouting capillaries, EC tube formation, and number of cells

## Conflict of interest

Cuifang Zhang, Helen He, Shuyao Yin, Mingyi Gao and Li Yu declare that they have no conflict of interest.
